# Gold Nanomaterials for Imaging-Guided Near-Infrared *in vivo* Cancer Therapy

**DOI:** 10.3389/fbioe.2019.00398

**Published:** 2019-12-05

**Authors:** Yuanyuan Tian, Sheng Qiang, Lianhui Wang

**Affiliations:** ^1^Weed Research Laboratory, Nanjing Agricultural University, Nanjing, China; ^2^Key Laboratory for Organic Electronics and Information Displays & Jiangsu Key Laboratory for Biosensors, Institute of Advanced Materials, National Jiangsu Synergetic Innovation Center for Advanced Materials, Nanjing University of Posts and Telecommunications, Nanjing, China

**Keywords:** gold nanomaterials, *in vivo*, photothermal therapy, surface-enhanced Raman scattering, photoacoustic imaging

## Abstract

In recent years, tremendous efforts have been devoted into the fields of valuable diagnosis and anticancer treatment, such as real-time imaging, photothermal, and photodynamic therapy, and drug delivery. As promising nanocarriers, gold nanomaterials have attracted widespread attention during the last two decades for cancer diagnosis and therapy due to their prominent properties. With the development of nanoscience and nanotechnology, the fascinating bio-applications of functionalized gold nanomaterials have been gradually developed from *in vitro* to *in vivo*. This mini-review emphasizes some recent advances of photothermal imaging (PTI), surface-enhanced Raman scattering (SERS) imaging, and photoacoustic imaging (PAI)-guided based on gold nanomaterials *in vivo* therapy in near infrared region (>800 nm). We focus on the fundamental strategies, characteristics of bio-imaging modalities involving the advantages of multiples imaging modalities for cancer treatment, and then highlight a few examples of each techniques. Finally, we discuss the perspectives and challenges in gold nanomaterial-based cancer therapy.

## Introduction

Because of the high incidence and mortality of cancer, scientists have paid long term attention to the diagnosis and treatment of cancer. Conventional cancer therapies have clear side effects (Liu et al., 2019a), thus these shortcomings have prompted researchers to look for effective diagnostic strategies to struggle against cancer (Chen et al., 2016; Ju et al., 2019). With the development of modern technology and biological medicine, diagnosis, and treatment based gold nanomaterials are emerging to improve the therapeutic effect. Particularly, photothermal therapy (PTT) based on gold nanomaterials is a promising therapeutic modality, which can be combined with the advanced imaging modalities for the multi-functional therapeutic application (Huang et al., 2017; Wu et al., 2019).

Gold nanomaterials have been widely investigated as considerable biocompatible platforms for the biological field due to the advantages of simple synthesis, large surface area, adjustable optical property, and multiple surface modification (Conde et al., 2012; Jackman et al., 2017). Over the past decade, numerous fundamental reviews have comprehensively investigated the synthesis, size, optical properties, and modification of gold nanoparticles (Daniel and Astruc, 2004; Jain et al., 2008; Sardar et al., 2009; Sau et al., 2010; Jones et al., 2011; Cao et al., 2014; Chauhan and Mukherji, 2014; Singh et al., 2014; Rai et al., 2016; Amendola et al., 2017; Pareek et al., 2017), so these characteristics will be only briefly mentioned in this mini-review. Herein, the photothermal imaging (PTI), surface-enhanced Raman scattering (SERS), and photoacoustic imaging (PAI) guided *in vivo* cancer therapy are focused. What is worth noting is excellent photothermal effect, the localized surface plasmon resonance (LSPR) effect and enhanced electromagnetic field (SERS) of gold nanoparticles in the near infrared (NIR) region. Due to low absorption of water and hemoglobin, the NIR region (700–1,300 nm) is ideal for gold nanomaterials to have a deeper penetration depth in the tumor therapy and imaging (Bao et al., 2016). Gold nanomaterial can convert the absorbed light energy into overheating in the surrounding environment through non-radiative conversion owing to the desirable absorption cross sections and photothermal conversion efficiencies (η) in NIR region (Cao et al., 2019). Therefore, gold nanomaterials are considered as exogenous photothermal transduction agents for PTI and contrast agents for PAI, which can accumulate at tumor tissue via the enhanced permeability and retention (EPR) effect (Henry et al., 2016). Moreover, LSPR effect is a surface plasmon resonance phenomenon of the noble metal nanomaterials, which is heavily dependent on the composition, shape, size, and micro-environmental medium (Guo et al., 2015; Tian et al., 2016, 2018). In addition, SERS is generated from the strong phonon-electron interaction in the nanogaps (Girard et al., 2016). Thus, the Raman signals are enhanced by several orders of magnitude, and gold nanomaterials labeled with reporter molecules can be used as nanotags for *in vivo* imaging (Ding et al., 2016). In short, these gold nanomaterials having resonance peak within NIR (>800 nm), including gold nanorods, gold nanocages, gold nanoshells, and assemblies, can be used as photothermal agents, imaging agents, contrast agents, and therapeutic agents (Shanmugam et al., 2014). Table 1 presents some examples of *in vivo* imaging and therapeutic modalities based on different types of gold nanomaterials.

**Table 1:** Examples of different structural characteristics, optical properties, and *in vivo* imaging modality.

**Structure and surface**	**Size (nm)**	**Wavelength (nm)**	**Laser**	**Models**	**Imaging modalities/η**	**References**
Nanoshells/nanomatryoshkas, thiol-PEG	150/90	~800	2 W/cm^2^, 810 nm	TNBC tumor- bearing female mice	PTI, 39%/63%	Ayala-Orozco et al., 2014
Nanocages, PVP and RBC-membrane	71–89	810–817	1 W/cm^2^, 850 nm	4T1 tumor- bearing BALB/c mice	PTI	Piao et al., 2014
Nanoshells, thiol-PEG	~120	780–800	4.5 W, 810 nm	Prostate cancer- patients	PTI	Rastinehad et al., 2019
Nanospheres, Au–Cu_9_S_5_, PMHC_18_-PEG_5000_	~20	~1,100	0.6 W/cm^2^, 1064 nm	CT26 tumor- bearing mice	PTI, 37%	Ding et al., 2014
Nanospheres, Au-silica	~120		0.29 W, 785 nm	Female nude mice	SERS	Bohndiek et al., 2013
Linear gold nanospheres assemblies, rBSA-FA	71.6	~650	0.5 W/cm^2^, 808/785 nm	MCF-7 tumor-bearing mice	PTI, SERS	Xia et al., 2018
Nanorods, Au-Ag-silica, PEG	80–97	~585	3 W/cm^2^, 660 nm	Ovarian cancer xenograft model and RCAS/TVA GBM mouse	PTI, SERS	Pal et al., 2019
Nanovesicles (PEG-b-PCL)	145	~800	1 W/cm^2^, 808 nm	MDA-MB-435 tumor-bearing mice	PTI, PAI, 37%	Huang et al., 2013
Nanorods, PDL/IR775c layered silica	400	780	795/920 nm	Female FVB/n mice	PAI	Dhada et al., 2019
Nanorods, PNIPAM	320	760	808 nm	Prostate cancer- bearing mice	PAI	Chen Y. S. et al., 2017

**Figure 1:**
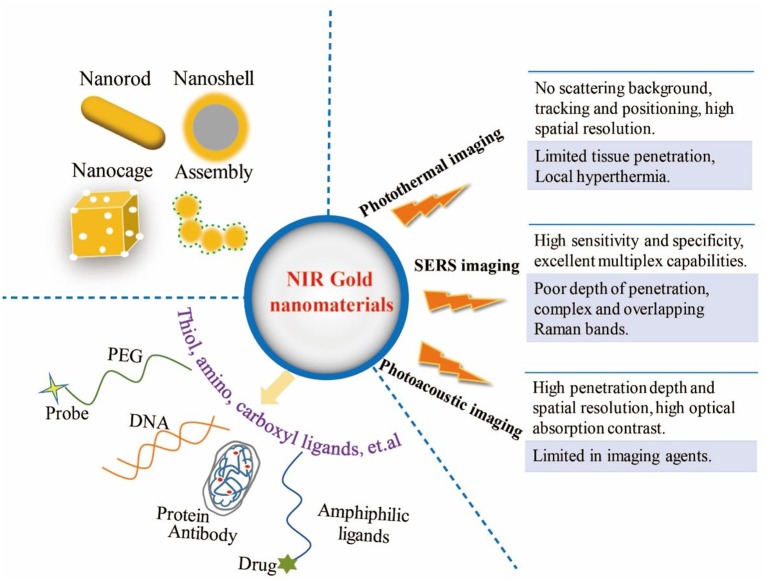
Illustration of multifunctional NIR gold nanomaterials platform for three imaging modalities.

After intravenous injection, considering the pH, high ionic strength and serum concentration in the complex biological environment of organism, surface functionalization of gold nanomaterials is essential to ensure the adequate repeatability and stability (Chen et al., 2010). Basically, modulation of the surface charge, biocompatible pH values, controllable biodistribution patterns, and better aqueous dispersion of gold nanomaterials could benefit from the surface modification approaches (Huckaby and Lai, 2018; Ruiz-Muelle et al., 2019). Furthermore, the rational surface modification strategies in anticancer application can also reduce the toxicity of nanomaterials, target effectively in the cancer tissue, increase the circulatory half-life, block absorption of serum proteins, avoid unexpected side effects, escape the clearance by the reticuloendothelial system and the liver and spleen macrophages (Otsuka et al., 2003; Kooijmans et al., 2016; Dai et al., 2018; Sztandera et al., 2019). Due to the facile surface chemistry properties, functional groups can combined with gold nanomaterials through weak interactions and the stable covalent anchors (Kou et al., 2009; Moraes Silva et al., 2016; Zou et al., 2016; Wang K. et al., 2017). So far, many modification approaches have been introduced by covering and ligand exchange, so gold nanoparticles can be modified with biopolymer (e.g., polyethylene glycol (PEG), oligonucleotides, antibodies, peptides) (Loh et al., 2016; Chen Y. et al., 2017; Anantha-Iyengar et al., 2019; Delpiano et al., 2019), hydrophobic drug molecules (e.g., paclitaxel, cisplatin, tamoxifen, doxorubicin) (Avitabile et al., 2018; Ma et al., 2018), biofunctional molecules (chitosan, silica, folic acid, polyunsaturated fatty acids, and hyaluronic acid, etc.) (Vigderman and Zubarev, 2013; Sztandera et al., 2019) and other amphiphilic ligands through functional bridges, such as thiol ligands (e.g., thiolate, dithiolate, thioctic acid), amino, and carboxyl moieties (Figure 1) (Daraee et al., 2014; Kong et al., 2017).

Based on the physical and optical properties of gold nanomaterials, the bioimaging techniques using gold nanomaterials are beneficial to enhance the accuracy of *in vivo* treatment and effectiveness of real-time monitoring. Recently, the non-invasive imaging-guided cancer therapy based on gold nanomaterials have been applied for providing more valuable information and improving the therapy outcomes. This mini review displays some examples of gold nanomaterials-based imaging modes in NIR region for *in vivo* diagnosis and therapy, including PTI, SERS, PAI, and their multiple imaging modalities. These characteristics of the three imaging modalities are shown in Figure 1.

## Photothermal Imaging (PTI)

For PTT, gold nanomaterials can transduce the absorbed light energy into heat energy for improving the temperature of tumor cells microenvironment. When the temperature exceeds 42°C, malignant cells are destroyed without damaging the adjacent healthy tissues (Qi et al., 2019). As a result, the therapeutic effect is far superior to that of laser irradiation alone (Abadeer and Murphy, 2016). The laser safety standard establish the maximum permissible exposure (MPE) values, which is on the basis of the damage threshold levels of laser light for skin (Jiao et al., 2019). For example, the MPE corresponding to 808 nm laser for skin is 0.33 W cm^−2^, and the MPE of 1,000–1,350 nm is 1 W cm^−2^ (Lin et al., 2017). Photothermal effect based on gold nanomaterials also afford a considerable imaging modality, that is, photothermal imaging (PTI), which has significant advantages over fluorescence imaging (Wang et al., 2009). PTI based on gold nanomaterials provides the accurate positioning, effective treatment, non-invasive therapy for various cancers in short time (Kang et al., 2018). Because it does not involve photobleaching or illumination saturation, and it is almost immune to background signals even accompanied with scattering (Boyer et al., 2002; Vines et al., 2019).

Conventional photothermal imaging is capable of detecting single molecules and differentiating gold nanoparticles, which is restricted by diffraction and resolution. A super-resolution photothermal imaging could be performed based on non-linear amplification signal for resolving multiple gold nanoparticles (Nedosekin et al., 2014). On account of the poor blood supply, tumors show reduced heat tolerance and thus can be destroyed in tens of minutes under hyperthermia condition (Qi et al., 2019). As known, intensive laser irradiation may lead to overheating and harm the nearby normal tissue. However, gold nanomaterials serve as the photothermal conversion agents to produce regional heating rapidly for PTT, and this method can effectively reduce irreversible tissue damage caused by laser irradiation (Ren et al., 2013). Owing to maximum transmittance in the blood and tissues in the NIR region, gold nanomaterials have deeper tissue penetration and lower background signal (Liu et al., 2014). With inherent advantages of multi-functionalization and tunable optical properties, gold nanomaterials coated with biomolecules had been developed to increase biological stability and prolong the circulation time *in vivo* (Choi et al., 2011). For example, red blood cell membranes coated gold nanocages (RBC-Au NCs) were combined with photothermal effects from gold nanocages and biological characteristics from RBCs, which showed good biological stability, observably improved tumor uptake, and longer retention time in blood (Piao et al., 2014). This work achieved 100% survival during 45 days and facilitated the RBC-Au NCs for the *in vivo* treatment with an improved efficacy using surface engineering.

Compared with other shapes, elongated nanoparticles have better affinity, greater diffusion and transmembrane rates, and they can penetrate the tumors rapidly and accumulate extensively (Lee et al., 2017). These had been demonstrated that the multifunctional nanomaterials had a high accumulation at tumor tissue in mice, which were able to kill cancer cells effectively by producing enough heat and reactive oxygen species. Chitosan/gold nanorod nanocarriers with loading anti-cancer drug cisplatin were prepared, which improved the therapeutic efficacy via synergistic effect of combining PTT and chemotherapy (Chen et al., 2013b). It had been found that the temperature of tumor site could reach 49°C in 10 min with IR irradiation and the tumor growth was almost completely suppressed by cisplatin-loaded nanospheres. As known, lanthanide ions have the ability to convert the NIR long-wavelength into visible wavelengths in photon upconversion process (Wang and Liu, 2009). Customarily, lanthanide doped nanocrystals having novel luminescent properties are considered as upconversion luminescence nanoparticles, which exhibit superior features in biological assays, such as low auto-fluorescence signals, narrow emission bandwidths, large anti-Stokes shifts, high penetration depth, and low toxicity (Huang et al., 2014; Gnach et al., 2015; Liu et al., 2019b). In order to increase the photothermal conversion efficiency and penetration depth in biological tissues, gold nanorods combined with rare-earth ions had been designed for PTT and multimodal imaging for anti-tumor therapy, because upconversion luminescence nanoparticles displayed deeper penetration and lower autofluorescence signals by anti-Stokes emission, and they could also transfer NIR light to gold nanorods via luminescence resonance energy transfer (Wang et al., 2018). With combination of phototherapy, gene therapy, and chemotherapy, the rational treatment strategy has an overwhelming superiority of tumor shrinkage and survival rate, which also could monitor host reactions and comprehensively evaluate tumor microenvironment. Lee et al. introduced silica-coated gold nanorods modified with rabies virus glycoprotein for treating brain tumors (Lee et al., 2017). The surface-modified nanomaterials could enter the brain through central nervous system and bypass the blood–brain barrier. The rod nanostructure increased the chance of interacting with the nicotinic acetylcholine receptor and the response to hyperthermia in NIR laser irradiation.

According to the reports, drug release, and chemotherapy displayed higher cytotoxicity for enhancing the therapeutic efficacy at raised temperatures from PTT (Vines et al., 2019; Wu et al., 2019). Indocyanine green was successfully sealed in chitosan/gold nanorod nanocapsule for PTT and photodynamic combined therapy (Chen et al., 2013a). Conde et al. adapted a triple-combination therapy that relied on drug-gold nanorods and siRNA-gold nanospheres for drug release, distinguish cancer and normal cells, and prevented cancer recurrence in a mouse model of colon cancer (Conde et al., 2016). In spite that drug-loaded nanocarriers with hyperthermia effect have therapeutic advantages for releasing targeting agents for tumor, there are still some drawbacks, such as insufficient or excessive dose by intravenous delivery (Tong et al., 2016; Wilhelm et al., 2016; Nabil et al., 2019). Recently, Lee et al. proposed a transplantable therapeutic interface based on a gold-coated nanoturf structure for on-demand hyperthermia therapy and drug delivery (Lee et al., 2018). The gold-coated polymeric nanoturf structure could not only serve as drug reservoir but also provide an induced heat under NIR irradiation, thus modulating drug releasing rate and controlling the surface temperature precisely for an esophageal cancer model. Moreover, to maximize the synergistic effects by exogenous and endogenous stimuli, two smart gold nanocages containers coated with photothermal and pH responsive polymer were designed for loading doxorubicin (Dox) and erlotinib (Erl), respectively (Feng et al., 2019). In the NIR irradiation and acidic tumor microenvironment stimulation, tumors were killed by synergistic therapy of PTT and time-staggered drug release strategy of Dox and Erl. The combination strategy obviously improved the therapy efficacy by controlling the order and continuance of drug in timing and spacing scale. These results show that it is difficult to completely ablate the tumor without any recurrence only based on PTI, because local photothermal is unable to impede the spread of cancer cells. Therefore, multiple imaging techniques need to be combined for redeeming the shortcomings of PTI.

In addition, gold nanoparticles are eliminated from the body to minimize health hazard at reasonable concentrations, which are supervised as medical devices by the Food and Drug Administration. Tremendous investigations on gold nanomaterials had been studied for biomedical sciences, and the preclinical safety of gold nanoshells had been established *in vitro* and *in vivo* (Gad et al., 2012). Recently, Halas et al. reported a study of photothermal ablation for prostate tumors in clinical trials which gold-silica nanoshells (7.5 mL/kg dose volume) were utilized in combination with imaging modality, without significant adverse events in 94% of patients (Rastinehad et al., 2019). This treatment protocol was demonstrated to be safe and feasible procedure for localized prostate cancer with low or intermediate risk, and it would open the door for gold nanomaterials in the clinical anticancer application. However, the bioinert and lack of biodegradation of gold nanomaterials bring uncertainty and toxicity to the human body. These negative feedbacks will directly affect the further clinical application of gold nanomaterials.

## SERS Imaging

In general, Raman reporter molecules are attached to the surface of gold nanomaterials, providing unique, and certain representative peaks as the signal source (Lu et al., 2010b). The inelastic scattering of gold nanotags has narrow characteristic bandwidths with several orders of amplification, which provides sufficient structural and quantitative information for biological interactions and analyte (Maiti et al., 2010). SERS imaging is being recognized as a promising optical modality for preclinical and clinical cancer application. Compared with fluorescent imaging, SERS imaging can avoid photobleaching and photoblinking and serve as an eligible alternative with good photostability for *in vivo* imaging (Pal et al., 2019). Normally, magnetic resonance imaging (MRI) is time-consuming imaging with low sensitivity. While SERS imaging is not only fast speed and super sensitivity, but also has excellent multiplex capabilities and fine specificity for multiplex targeting (Von Maltzahn et al., 2009). In recent decades, SERS-based detection with enhanced electromagnetic field is extremely sensitive for trace analysis in nanoscale regions (Bardhan et al., 2014). More importantly, SERS imaging has weak interference signals in biological tissues, thus it is popular in high-sensitive cell tracking (Wang Z. et al., 2017).

As known, MRI has been usually used for preoperative directions and determining the macroscopic profile of the tumor. However, the typical imaging methods were limited by low sensitivity, and spatial resolution, and it was particularly difficult to describe the actual margins of tumors due to the brain shift in surgery (Orringer et al., 2010). For preoperative evaluation and intraoperative treatment, Kircher et al. showed a triple-modality to delineate the brain tumor boundaries in living mice (Kircher et al., 2012). Ideally, SERS imaging is a type of real-time imaging technique, which plays a main role in navigation for accurately delineating the brain tumor margins and guiding the tumor resection. With the development of SERS imaging for tissue in small animals, an imaging system with large area and high spatial resolution had been exploited (Bohndiek et al., 2013). This unique spectroscopic instrument showed an ultra-high sensitivity for a non-invasive imaging modality. It simplified animal handling, and thus could realize rapid and multiplex detection for the characterization of uptake dynamics *in vivo*. Gold nanoparticles covered with Raman active molecules are contrast agents with an enhanced scattering intensity. The size of core nanomaterials, the properties of Raman molecules, and the number of the molecules absorbed on the surface determine the order of magnitude of amplification. Harmsen et al. presented an administrative SERS nanoparticle for intraoperative imaging in mouse models of glioblastoma, which was composed of a gold core and a layer of Raman reporter molecules embedded in a silica shell (Harmsen et al., 2015). As a result, the limit of detection (LOD) reached femtomolar. Furthermore, Wang's group synthesized 3D flower-like hierarchical gold nanostructures and gold nanostars for cancer therapy (Song et al., 2016, 2018, 2019). The SERS-active gold nanomaterials could use as SERS tags and nanocarriers for cell imaging and drug delivery, which were suitable candidates for promising SERS-imaging.

For *in vivo* SERS mapping, gold nanostars conjugated with antibodies and Raman tags were able to detect the immunomodulators and immunomarkers simultaneously (Ou et al., 2018). With the accumulation of gold nanostars by intravenous injection, the real-time longitudinal tracking of the both biomarkers was implemented, and the sensitivity and specificity of the relevant SERS signals displayed different levels in breast cancer tumors. Furthermore, the high-resolution SERS imaging could evaluate the distributions of gold nanostars in tumors which were closely related to vascular density. With wonderful optical properties, one-dimensional gold nanoparticle assemblies (GNAs) with good dispersion, stability, and biocompatibility were developed for *in vivo* imaging (Xia et al., 2018). The GNAs exhibited numerous ultra-small nanogaps (smaller than 1.0 nm) and flexible caterpillar-like nanostructures, which could change their morphology randomly. Due to the remarkable SERS signal and good photothermal effect, the GNAs were used as an efficient platform for SERS imaging and photothermal imaging. Currently, SERS scanners are dependent on the point-by-point acquisition, with a relatively slow speed, which could not be satisfied for real-time and rapid imaging in oncological application. Encouraged by the versatility of fluorescence imaging and the specificity of SERS detection, rational design of nanoprobes had been carried out for fluorescence and SERS imaging in ovarian cancer xenograft models (Pal et al., 2019). The bimodal nanoprobes based on DNA and gold nanorods with three consecutive layers were used successfully for imaging-guided tumor ablation and PTT. Meanwhile, aptamer-conjugated gold nanocage has also been built as the bifunctional theranostic platforms for SERS imaging and NIR-triggered PTI (Wen et al., 2019). However, the real biological environment of the human body is more intricate than animal models, and most of the modified nanoprobes are likely to be metabolized directly by immune system. Therefore, the results can lead to the reduced active targeting, the limitation of circulation time and insufficient dose of these nanoprobes that will affect the therapeutic and imaging effects.

## Photoacoustic Imaging (PAI)

As known, contrast agents or tissue can absorb the non-ionizing laser and be heated, leading to a transient thermoelastic expansion. Subsequently, wideband acoustic waves are produced as photoacoustic waves, which can be captured on the surface of target substance (Ju et al., 2019). Usually, computed tomography (CT) imaging is high toxicity, the non-specific distribution of contrast agents, and short imaging time (Xu et al., 2019). However, PAI relies on the photoacoustic effect with high reliability, and the gold contrast agents can be purposely modified. Additionally, compared with MRI and fluorescent imaging, PAI is non-invasive, quantitative, and speedy, and it also presents high spatial-resolution of ultrasound imaging and contrast of optical imaging (Zackrisson et al., 2014). PAI has been demonstrated as a powerful tool to visualize biological tissues with the advantages of deep penetration and high spatial-resolution (Agarwal et al., 2007).

Gold nanomaterials are one kind of contrast agents for providing improved photoacoustic signals because of chemical inertness and large absorption cross sections in NIR region (Yang et al., 2015). PEG-coated Au nanocage and pegylated hollow gold nanospheres had been described as optical contrast agents for *in vivo* PAI (Yang et al., 2007; Lu et al., 2010a). Specifically, PEGylation-modified gold nanostructures with different shapes, including nanospheres, nanodisks, nanorods, and cubic nanocages, had already been investigated for bioactivity analyses in EMT6 breast cancer model (Black et al., 2014). Furthermore, gold nanorods had been applied to image in ovarian tumor models with a multimodal imaging (Jokerst et al., 2012a). In this study, the parallel PAI and SERS imaging had complementary capabilities, where the PAI characterized the size, morphology, and stage of the tumor, and SERS imaging guided the surgical resection. The silica-coated gold nanorods as PAI agents exhibited a higher cellular up-take and had no toxic effect on normal cells. They had been prepared to quantitate and image mesenchymal stem cells for living mice in real time, and the results showed the spatial resolution of 340 μm and the temporal resolution of 0.2 s (Jokerst et al., 2012b). To track the stem cell in cardiovascular diseases, silica-gold nanorods with coating IR775c, which was a sensitive dye of reactive oxygen species, had been developed for PAI (Dhada et al., 2019). The nanoprobe had a high spatial and temporal resolution, and they displayed a 5% viability of mesenchymal stem cells after 10 days.

Due to the small size of nanoparticles with the overlap heating volume, the intensity of photoacoustic signal could be increased proportionally to the huge thermal energy which was rapidly generated from the conversion from optical absorption (Chen et al., 2012). Chen et al. synthesized small gold nanoparticle with controlled aggregation in a volume-changing nanocarrier that had photothermal stimuli-responsive behavior (Chen Y. S. et al., 2017). Overall, the PAI shows a dynamic contrast-enhancement, while the background signals from tissue is suppressed. Although the photonic nanoclusters are well-understood and have been applied in a variety of bioimaging, the bottom-up assembly based on nucleic acid scaffolds is still a challenge. As an example, the plasmonic self-assembles had been exploited as photo-responsive probes for multimodal SERS and PAI *in vivo* (Köker et al., 2018). In this method, the discrete gold nanoparticles were functionalized with two complementary split fragments of green fluorescent protein. Park et al. manufactured a gold nanoclusters based on gold nanoparticles (about 4.5 nm) and albumin for optically visualizing and treating colon cancers via PTT, fluorescence imaging, and PAI (Park et al., 2019). By optimizing the size, shape, and optical absorption of the hybrid albumin nanostructures, the promising platform displayed a strong hyperthermic effect, as well as a balance between LSPR and fluorescence resonance energy transfer effects.

## Conclusions and Perspectives

The research on bioimaging has been mainly promoted by the systematic exploitation of nanomaterials and the combination of modern techniques. Different types of functionalized gold nanosensors have been reported with the development of the surface modification. In this review, the multifunctional gold nanomaterials have been showed for imaging-guided *in vivo* cancer therapy using photothermal, SERS, PAI, and multiples imaging modalities. Various types of gold nanostructured loading platforms have been studied for imaging and treatment of cancers. Nonetheless, there remain some challenges and opportunities for further and higher demand for *in vivo* and clinical studies. Firstly, the development of gold nanomaterials is crucial for the applications of optical imaging and synergetic therapy. For ideal imaging and therapeutic results, three-dimensional structure, special configuration of assemblies and composite gold nanomaterials are popular for the bio-application. For example, some gold nanomaterials with well-defined architectures and luxuriant hot spots, including composite gold nanomaterials and self-assemblies, may generate stronger electromagnetic-field enhancement for SERS imaging. These novel nanomaterials also can be explored as excellent contrast agents for PAI to overcome the problem of penetration depth. Meanwhile, the multifunctional agents and biological responsive (acceerative or passivated) molecules also necessary to be developed with less toxic and better dispersion for the organism. Therefore, the appropriate multifunctional agents can avoid the possibility that the partial malignant cells are intact after local hyperthermia in the effective PTT. It is also possibility to enhance the NIR responsiveness, improve the accuracy of targeting, and increase the efficiency of the delivery during the cancer treatment. On the other hand, SERS possess single molecule sensitivity *in vitro* experiment, and the elaborated gold nanomaterials will be promising candidate to reach this level and free from the interference of other species in the biological system. Consequently, efficient composite gold nanoparticles with desirable surface modification agents are pressingly needed to achieve the desired effect *in vivo*. Similarly, the thermal resistance also can be reduced by modifying interfacial agents to improve the photoacoustic signal and resolution. Almost all of the experimental and theoretical researches have focused on the animal modes, while the clinical study is still lacking. Lastly, it is necessary to integrate diagnosis, multimodal imaging, and enhanced therapies for clinical application. Elaborate detecting strategies and sensitive multiplex techniques can enhance the spatial and temporal resolution, capture actual dynamic processes in real-time, and answer the fundamental biological questions in modern medicine. Finally, most of the research has focused on the biological interactions, intracellular distribution, and the transport behaviors of gold nanomaterials, while the detailed mechanism of different interaction with biomolecules and the process of internalization are still lack of deep understanding.

## Author Contributions

LW and SQ designed and revised this maunscript. YT wrote this manuscript.

### Conflict of Interest

The authors declare that the research was conducted in the absence of any commercial or financial relationships that could be construed as a potential conflict of interest.
